# Supplementary material for: F-actin architecture determines the conversion of chemical energy into mechanical work
Source: Nat Commun. 2024 Apr 24;15:3444. doi: 10.1038/s41467-024-47593-x (PMC11043346; doi:10.1038/s41467-024-47593-x)
Supplement: Supplementary file 1 — Supplementary Information [file 41467_2024_47593_MOESM1_ESM.pdf]

Supplementary Information for

F-actin architecture determines the conversion of chemical energy into mechanical work

Ryota Sakamoto and Michael P. Murrell*

*Corresponding author: **Email**: michael.murrell@yale.edu

This PDF file includes:

Supplementary Notes 1-3

Figures S1 to S14

Supplementary References 1-20

Supplementary Note 1: Additional contributions to the mechanical power estimation

The mechanical power estimates in the present work are solely based on the change in the actin network volume. Consequently, there are possible additional contributions to the mechanical work performed by myosin. In the following, we discuss the limitations of the mechanical work estimation method we employed and the potential contributions to mechanical work that were not considered in our estimates.

In our estimation, we used the network volume change as a first-order readout of the mechanical work performed by myosin-generated stress. We referred to the energy stored in the volume variation mode of deformation as $E_{\Delta V}$. However, there are other possible contributions to the energy cost that exist during the contraction of the network. In this case, the total energy cost of deformation is expressed as $E_{\text{total}} = E_{\Delta V} + E_{\Delta V=0} + E_{\text{dissipative}} + E_{\text{load/architecture}}$, where $E_{\Delta V=0}$ represents the energy stored in volume conservation modes of deformation (e.g., filament bending/stretching), $E_{\text{dissipative}}$ is the dissipated energy during contraction (e.g., filament breakage), and $E_{\text{load/architecture}}$ is the contribution of load-dependent/architecture-specific stresses generated by myosin motors. In the following, we discuss how these missing energies contributes to our estimates of mechanical work.

- *Contribution of a volume-conserving compression mode.* First, fraction of energy could be stored in volume conservation modes of deformation ($E_{\Delta V=0}$) such as filament buckling. To evaluate the contribution of volume conservation modes, we estimate the buckling energy of F-actin. With a bending stiffness of $k = 7.3 \times 10^{-26}$ N m^2 (1), and a filament length $L = 1$ µm, the corresponding energy to buckle is $E_b = kL/2r_c^2 = 4.1 \times 10^{-19}$ J (1,2), where $r_c = 300$ nm is the radius of curvature of buckled filaments measured in the contracting actomyosin network (3). To estimate the maximum contribution of the filament buckling, we assume that the total 6 µM actin (in our experimental condition) forms $L = 1$ µm F-actin and buckles. Since 1 µm F-actin consists of ~370 monomers (4), 6 µM actin in a droplet with radius $R = 20$ µm is equivalent to a total number of 3.2×10^5 F-actin with $L = 1$ µm. Assuming all the F-actin is buckled within 10 min during the contraction of the network, the maximum energy used to buckle the filaments per unit time is $\sim 2 \times 10^{-16}$ J s^{-1}. This value is two orders of magnitude smaller than the maximum power calculated solely based on the volume variation mode in the main text ($\sim 1.5 \times 10^{-14}$ J s^{-1}). Thus, the energy used for the buckling of F-actin is not significant compared to the volume variation modes of mechanical power. Although the exact contribution of crosslinking and bundling to the volume conservation modes is not fully understood, we expect that these effects will stiffen the F-actin, making filaments less likely to buckle, and the contribution of architecture will not be significant.

- *Contribution of a volume-conserving shear mode.* In addition to the compression that leads to buckling, stretching contribution of actin exist in the volume conservation modes of deformation ($E_{\Delta V=0}$). To estimate the maximum contribution of the elastic energy stored in the F-actin by stretching, we assume that the total 6 µM actin (in our experimental condition) forms $L = 1$ µm F-actin, in which they are stretched until breakage by reaching the critical tensile stress of filament breaking $F_c \sim 500$ pN (5). We consider the enthalpic stretching

regime as the network is strongly contracted, in which filaments behave similar to a rigid rod (linear spring), respond to longitudinal tensile stresses by increasing their contour length (6-8). The extension of the F-actin with $L = 1$ μm under tensile stress of $F_c \sim 500$ pN is estimated to be $\Delta L = F_c/K \sim 11$ nm, using a value of the stiffness $K = 43.7 \times 10^{-3}$ N/m of a single actin filament with $L = 1$ μm (9). It should be noted that a complete model considering both entropic and enthalpic stretching estimated $\Delta L \sim 4$ nm at tensile stress of ~500 pN (8). Using the stiffness $K = 43.7 \times 10^{-3}$ N/m (9) and the extension length $\Delta L = 11$ nm, enthalpic stretching energy of F-actin yields $K\Delta L^2/2 \sim 2.6 \times 10^{-18}$ J. Using the total number of 3.2×10^5 F-actin with $L = 1$ μm in a droplet with radius $R = 20$ μm, the total maximum energy stored in the stretching mode per unit time during 10 min of contraction is estimated to be $\sim 1.4 \times 10^{-15}$ J s^{-1}. This value is one order of magnitude smaller than the maximum power calculated solely based on the volume variation mode in the main text ($\sim 1.5 \times 10^{-14}$ J s^{-1}). Thus, the energy used for the stretching of F-actin is not significant compared to the volume variation modes of mechanical power. We note that the presence of the critical forces of filament breakage limits the contribution of the energy stored in the stretching mode, in which torsional strain imposed on the F-actin during contraction may decrease the critical breakage forces (5), thereby the contribution of the stretching mode could be further reduced.

- *Contribution of a dissipated energy during contraction.* Secondly, not all the energy is stored in elastic energy, but dissipative effects such as plastic reorganization and fracture/severing of the network occur ($E_{\text{dissipative}}$) (3,10,11). To evaluate the contribution of the dissipative effects, we estimate the energy used to sever the filaments. The tensile stress required to break/sever a filament under tension is 500 pN (5). To separate the actin monomers from each other with the typical spacing between monomers, 2.7 nm (12), corresponds to the energy cost of severing a filament as $\sim 1.35 \times 10^{-18}$ J. To estimate the maximum energetic cost of severing, we utilized the observation that filament buckling coordinates the severing, where severing predominantly occurs below a curvature radius of buckled filaments ~300 nm irrespective of the cross-linking density (3). Assuming a total number of 3.2×10^5 F-actin with $L = 1$ μm is buckled and severed within 10 min during the contraction of the network, the maximum energy used to severe the filaments per unit time leads $\sim 7.2 \times 10^{-16}$ J s^{-1}. This value is two orders of magnitude smaller than the maximum power calculated solely based on the volume variation mode in the main text ($\sim 1.5 \times 10^{-14}$ J s^{-1}). Thus, the contribution of dissipative effects due to severing of F-actin is not significant compared to the"volu'e variation modes of mechanical power. When the network is crosslinked, severing could be enhanced 2-3 times (3), but even so, the energy dissipation due to severing is still less than the volume variation modes. Although the exact contribution of crosslinking and bundling to the dissipative modes is not fully understood, we expect that these effects will stiffen the F-actin, making filaments less likely to be severed, and the contribution of severing will not be significant.

- *Contribution of load-dependent/architecture-specific stresses.* Furthermore, there is potential need for corrections in myosin-based stress generation depending on different architectures ($E_{\text{load/architecture}}$), as we rely on the duty ratio of unloaded myosin and did not consider the contribution of architectures, such as the polarity

of bundles, to myosin force generation. For example, previous experimental and numerical simulation studies have shown that the polarity of actin bundles crosslinked by fascin or fimbrin influences the load-dependent myosin stress generation (13). It was demonstrated that myosin moves persistently in unipolar bundled networks crosslinked by fascin, whereas myosin motion is confined under mixed polar bundled networks crosslinked by fimbrin. This is because myosin binding to opposing F-actin in the bundle resists the myosin movement, resulting in the buildup of a force dipole. Using numerical simulations considering the load-dependence of myosin force generation and polarity of bundles, it was shown that the forces applied to the bundled networks vary by ~4 times at maximum depending on the polarity of bundles for myosin thick filaments composed of 250 heads. In our estimates of mechanical work, we have shown that unipolar bundles produce ~10 times larger mechanical power compared to mixed polar bundles ($\sim 1.2 \times 10^{-14}$ J s^{-1} in unipolar; $\sim 1.2 \times 10^{-15}$ J s^{-1} in mixed polar). Therefore, even if we consider the correction of 4 times larger myosin stress in mixed polar bundles, the mechanical power of unipolar bundles is still larger. Hence, the contribution of load/architecture-dependent myosin force generation does not alter the conclusion of our manuscript.

For branched architectures, it was experimentally and numerically shown that larger branching reduces stress propagation due to the inhibition of myosin motion, where a highly branched actin network with many barbed ends pointing outward from the mother filaments could make it difficult for myosin thick filaments to walk or slide (14). In this case, the effective stress applied to myosin is decreased, thus the correction to the stress leads to lower mechanical power. In the main text, we argued that the mechanical power generated within Arp2/3 branched network is reduced compared to control and linear F-actin architecture; thus, the correction to the branched architecture further signifies the influence of branching in our estimates of mechanical power. Overall, the contribution of load/architecture-dependent myosin stress generation will not change the main conclusion of this work.

In summary, our estimates of myosin-based mechanical work do not encompass: 1. Volume conservation modes, 2. Dissipative effects, 3. Load/architecture-dependent myosin force generation. As a result, our estimates provide a lower bound; yet, considering these additional contributions will not alter the main conclusion.

Supplementary Note 2: Potential contributions of the bending rigidity of F-actin to mechanical work

The rigidity of actin networks may change with architecture, as previously reported by rheological measurements (the elastic modulus of the Arp2/3-nucleated networks ~1 kPa (15), actin networks ~10 Pa; fascin-crosslinked networks ~50 Pa; α-actinin-crosslinked networks ~100 Pa (16); fimbrin-crosslinked network ~50 Pa (17); note that elastic modulus of the crosslinked networks were measured at ~1 mol% crosslinkers to actin, so that the absolute elasticity can be much higher in the present system). The more rigid the filaments are, the less bent they become, and mechanical work is more linearly applied during contraction, allowing to neglect nonlinear effects such as filament bending and severing. Consequently, our estimates of myosin-based mechanical work, calculated based on volume change, could be more relevant for the contraction of a more rigid actin architecture.

It is important to note that our focus is on how the F-actin architecture alters myosin contractility, rather than the rigidity of the filament itself. Specifically, we aim to compare branched and linear F-actin architecture nucleated by Arp2/3 and mDia1. For this purpose, the nucleator concentrations were chosen to be high enough to alter the contractile behavior of the actomyosin network as reported in the previous study (14). In the case of crosslinkers, architectural differences arise from the polarity of actin filaments in bundles (fascin is parallel; fimbrin is antiparallel) or filament spacing (fimbrin ~8 nm; α-actinin ~35 nm), not the difference in filament rigidity. For this purpose, crosslinker concentrations were chosen to be high enough to alter the motion of myosin thick filaments (13). Therefore, we have chosen the high enough concentrations of the crosslinkers and nucleators to impact myosin force generation via F-actin architecture.

Supplementary Note 3: Detailed Experimental Methods

Protein purification

Purified proteins were prepared by following the method described in a previous study (14,18). Briefly, SNAP tagged mDia1(FH1C) containing a carboxy-terminal His(6x)-tag was expressed in E.coli (BL21-Codon Plus (DE3)-RP strain). The cells were induced with 500 μM IPTG at 16 °C and purified using Talon® metal affinity resin by following the manufacturer's instructions. The mammalian SNAP-mDia1 (FH1C)-HIS plasmid was a gift from David Kovar lab. N-WASP fragment WWA (aa400–501, also called VCA) containing a carboxy-terminal His(6x)-tag was expressed in E.coli (BL21-Codon Plus (DE3)-RP strain). Fimbrin (full-length; pET-21a-Fim1p-His) was expressed in E.coli (BL21-Codon Plus (DE3)-RP strain). The cells were induced with 1 mM IPTG for 2 h at 37 °C and purified using Ni-NTA resin by following the manufacturer's instructions, followed by another purification using the size exclusion chromatography.

Fluorescent labeling of skeletal muscle myosin

Skeletal muscle myosin (rabbit, MY02; Cytoskeleton Inc.) is labeled with an Alexa Flour 647 nm C2 Maleimide in reduced conditions by following the previous study (1,2). Briefly, myosin is reduced in labeling buffer (50 mM HEPES (pH 7.0), 0.5 M KCl, 1 mM EDTA, 10 mM DTT, pH 7.6) then dialyzed in DTT-free labeling buffer overnight. The dialyzed myosin solution is centrifuged to remove insoluble matter and the supernatant is labeled with Alexa Fluor C2 Maleimide, which is added in 5-fold molar excess of myosin. The reaction is performed for 1 h at 4 °C and quenched with the addition of 1 mM DTT. Labeled myosin are collected after running through a desalting column (Pierce, 5 K MWCO, 5 mL). Optical absorbance is measured at 280 nm and 647 nm to determine the degree of labeling.

Supplementary Figures

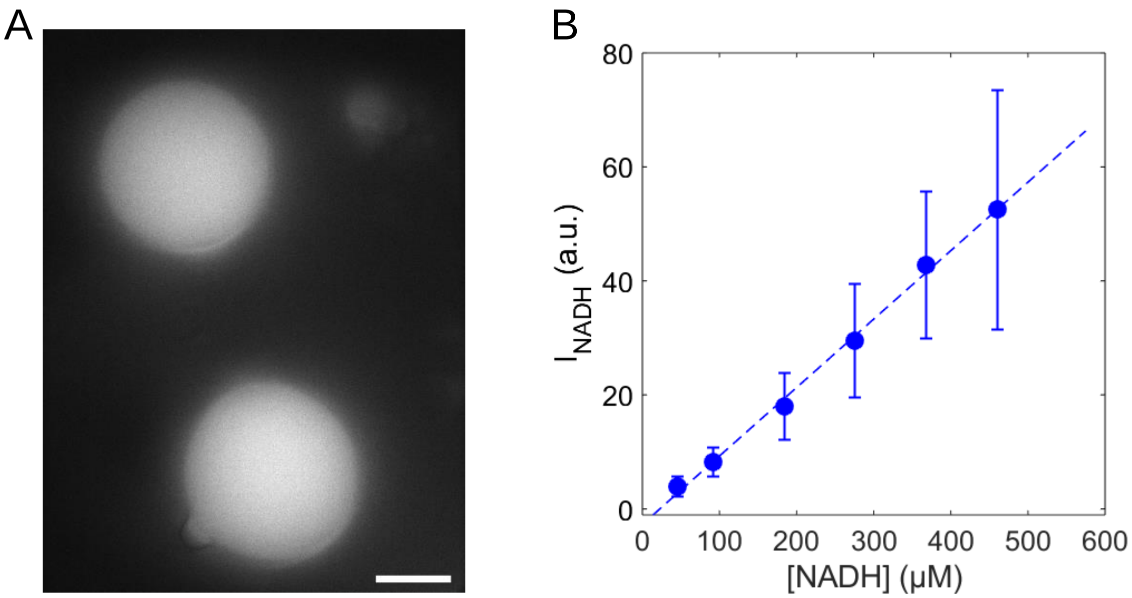

Supplementary Figure 1. Calibration curve for converting NADH fluorescence to NADH concentration. (**A**) The entire non-cropped field of view in confocal microscopy. The single region of interest typically contains 1-3 droplets, which ensures that droplets are adequately separated to prevent coalescence during measurements. Scale bar, 20 µm. (**B**) Droplets containing known concentrations of NADH were analyzed (n=558 droplets and N=6 independent experiments). Data points are mean +/- SD. The dashed line is the linear fitting, $I_{\mathrm{NADH}} = 0.1173 \times [\mathrm{NADH}]$.

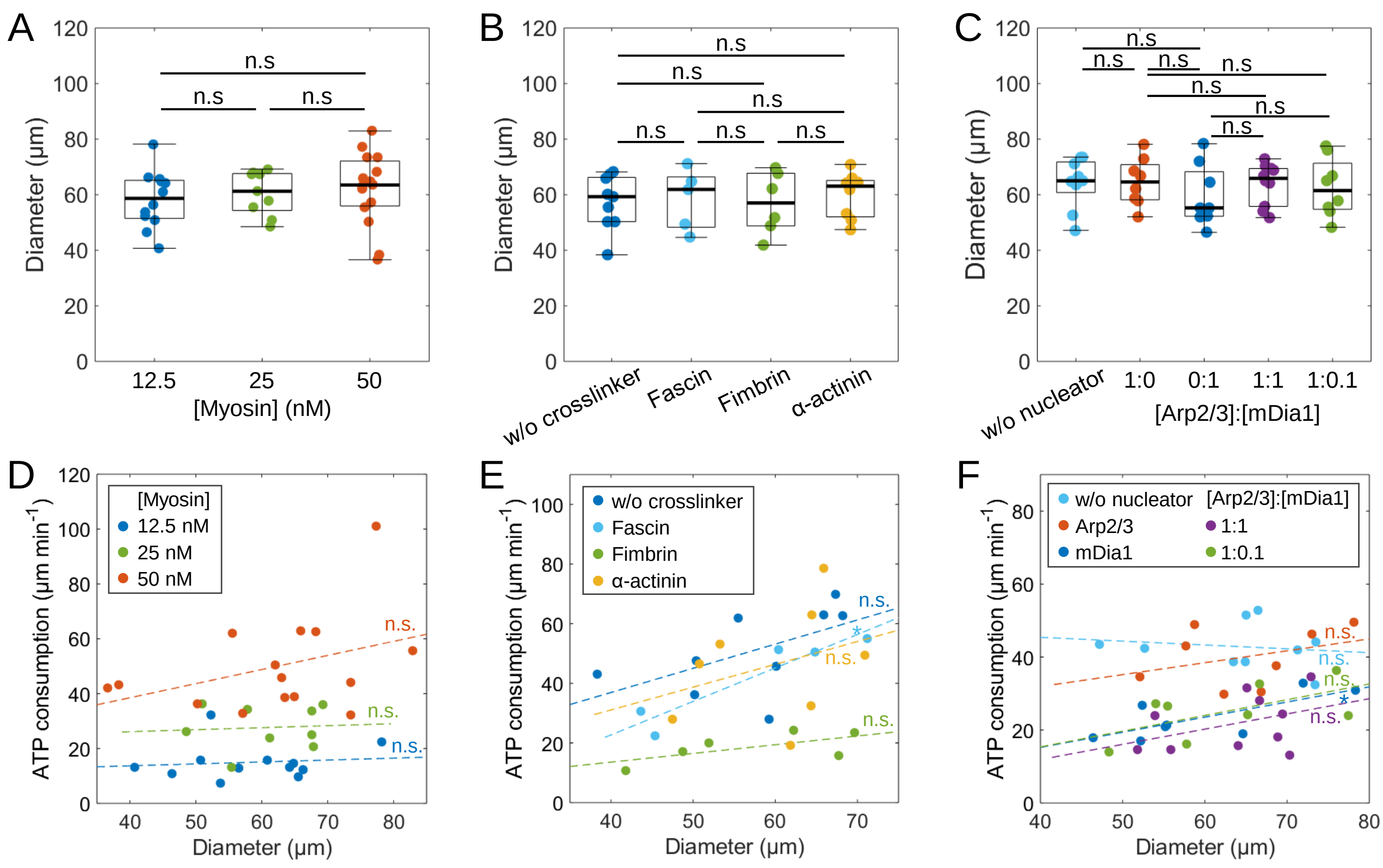

Supplementary Figure 2. The analyzed droplet size was comparable in different conditions. (**A**) Boxplot showing the droplet size analyzed for ATP consumption rates at different myosin concentration (n=12 droplets and N=6 independent experiments for 12.5 nM; n=9 and N=5 for 25 nM; n=15 and N=8 for 50 nM). (**B**) Boxplot showing the droplet size analyzed for ATP consumption rates in various crosslinkers (n=9 and N=4 in without crosslinkers; n=5 and N=3 in fascin; n=6 and N=3 in fimbrin; n=8 and N=4 in α-actinin). (**C**) Boxplot showing the droplet size analyzed for ATP consumption rates in various nucleator compositions (n=9 and N=5 in without nucleators; n=8 and N=5 for Arp2/3 only; n=8 and N=4 in mDia1 only; n=10 and N=5 in [Arp2/3]:[mDia1] = 1:1; n=8 and N=4 in [Arp2/3]:[mDia1] = 1:0.1). (**D**) Scatter plot showing the droplet size vs ATP consumption rates at different myosin concentrations. (**E**) Scatter plot showing the droplet size vs ATP consumption rates in various crosslinkers. **(F**) Scatter plot showing the droplet size vs ATP consumption rates in various nucleator compositions. *: $p<0.05$. n.s.: not significant.

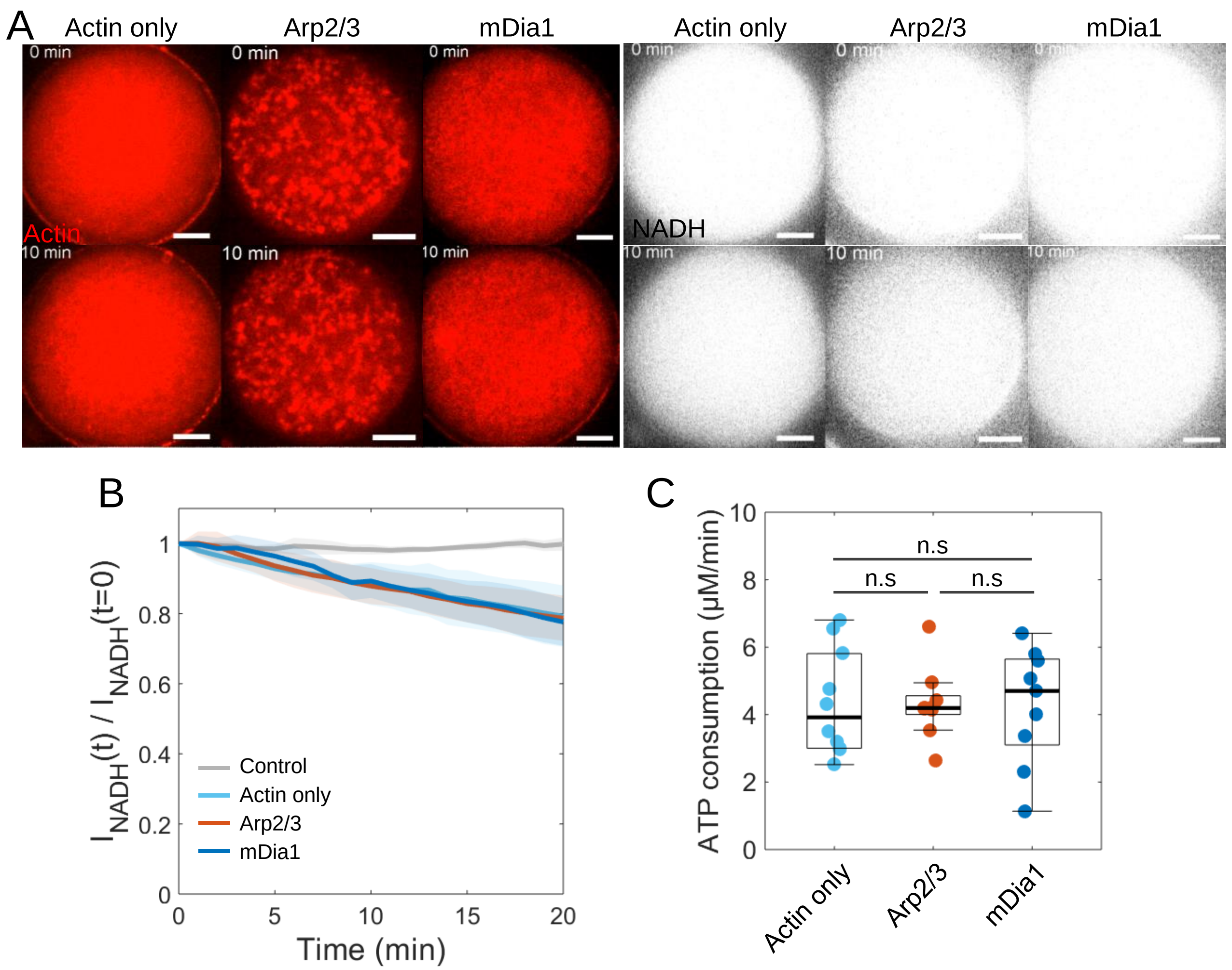

Supplementary Figure 3. ATP consumption rate of actin. (**A**) Snapshots showing the actin fluorescence (top) and NADH fluorescence (bottom) within droplets containing actin and actin nucleators, Arp2/3 or mDia1, without myosin. (**B**) Normalized NADH fluorescence over time (n=11 droplets and N=3 independent experiments in actin only; n=9 and N=4 for Arp2/3; n=9 and N=3 for mDia1). Curves are mean$\pm$std. (**C**) Boxplot showing the ATP consumption rate for different actin nucleators. There is no significance for all combination (*p*-value > 0.05, n.s.). Scale bars are 10 µm.

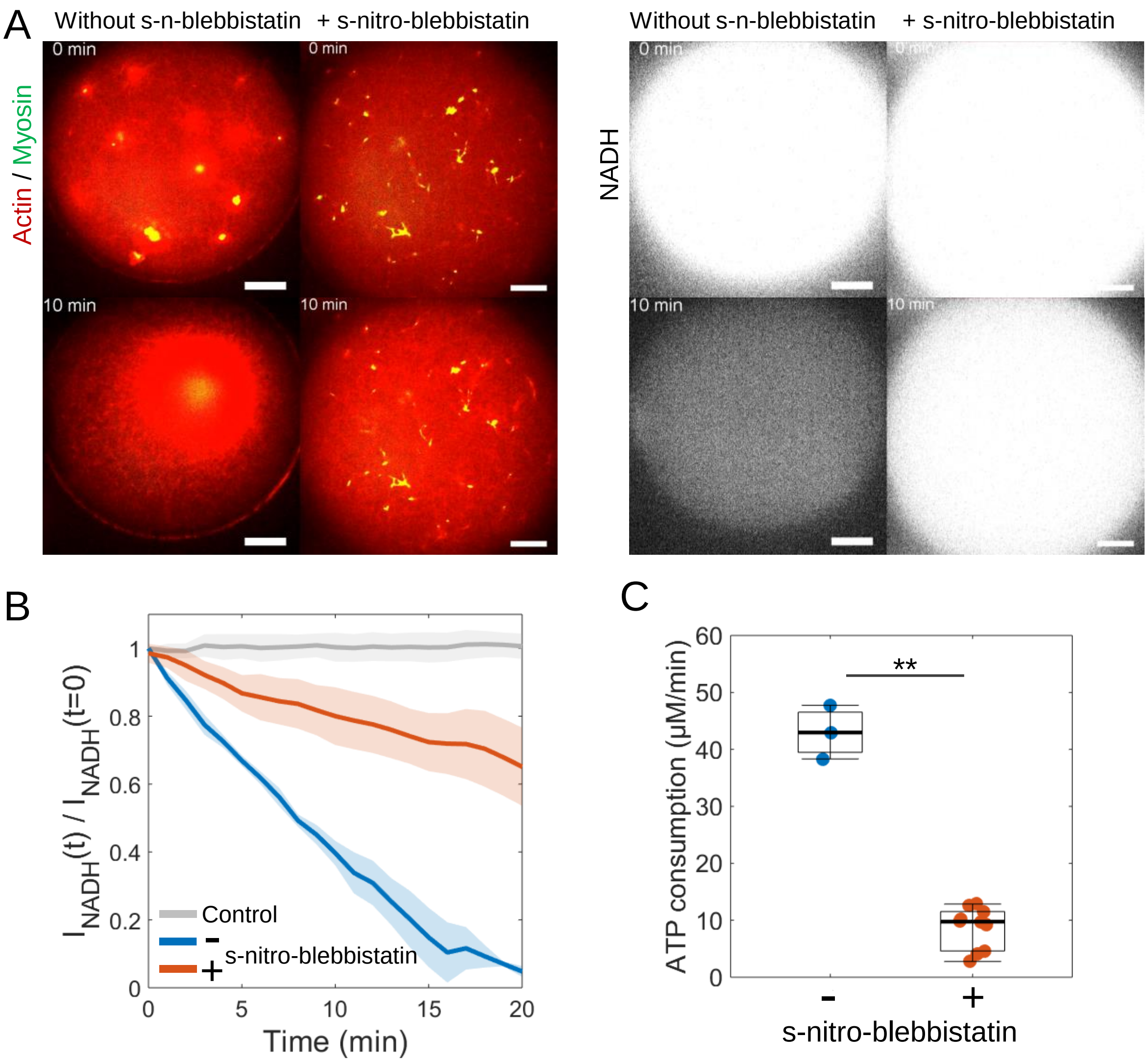

Supplementary Figure 4. ATP consumption rate under inhibition of myosin ATPase activity. (**A**) Snapshots showing the actin and myosin fluorescence (left) and NADH fluorescence (right) within droplets in the presence of 1 mM s-nitro-blebbistatin (Movie S2). Myosin concentration is 50 nM. (**B**) Normalized NADH fluorescence over time (n=12 droplets and N=6 independent experiments in control; n=3 and N=3 in without s-nitro-blebbistatin (-); n=10 and N=10 in with s-nitro-blebbistatin (+)). Curves are mean±std. (**C**) Boxplot showing the ATP consumption rate in B. ** represents $p<0.01$. Scale bars are 10 μm.

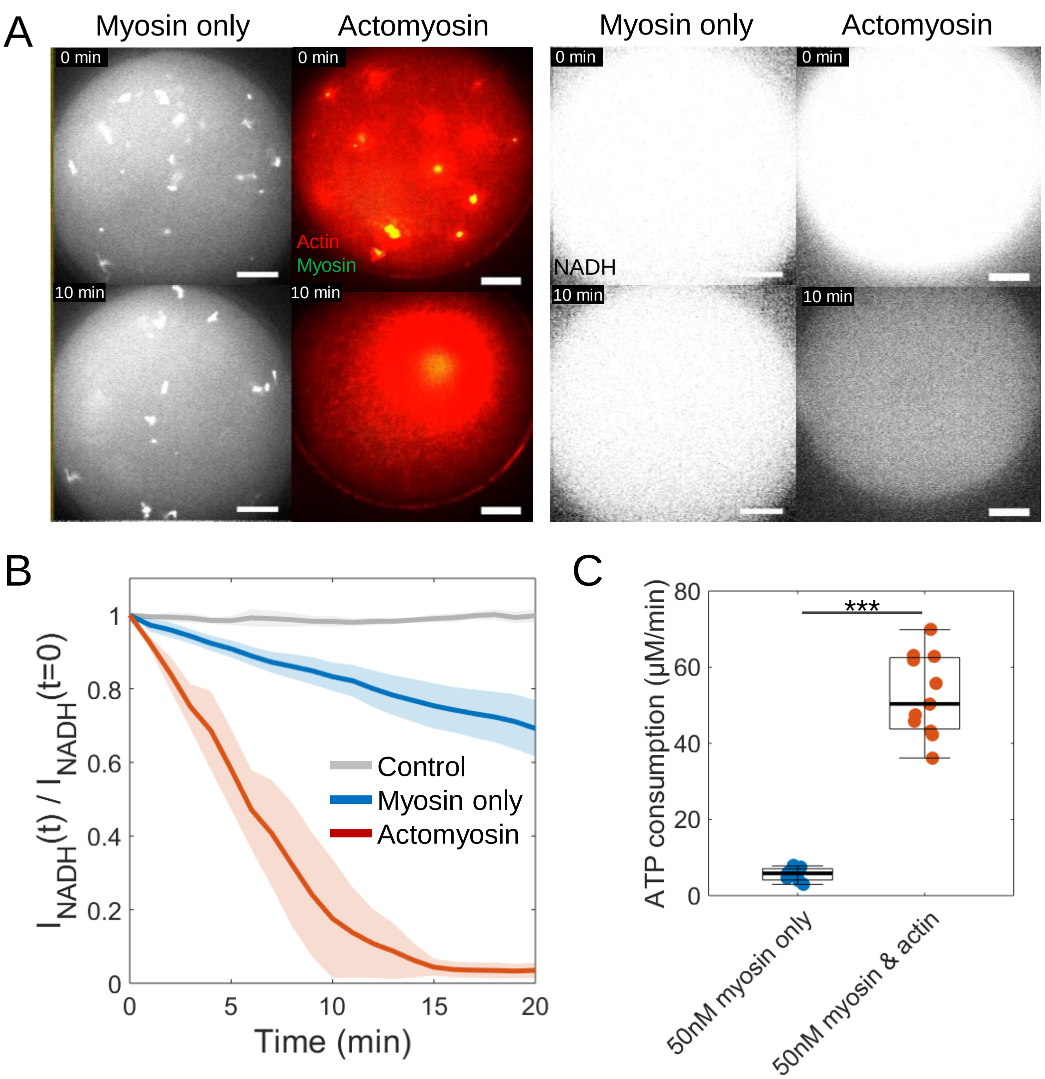

Supplementary Figure 5. ATP consumption of myosin in the absence of F-actin. (**A**) Snapshots showing the actin and myosin fluorescence (left) and NADH fluorescence (right) within droplets only containing myosin or actomyosin. Myosin concentration is 50 nM. (**B**) Normalized NADH fluorescence over time (n=7 droplets and N=3 independent experiments for myosin only; n=11 and N=4 for actomyosin). Curves are mean±std. (**C**) Boxplot showing the ATP consumption rate for droplets with myosin only or actomyosin. *** represents $p<0.001$. Scale bars are 10 μm.

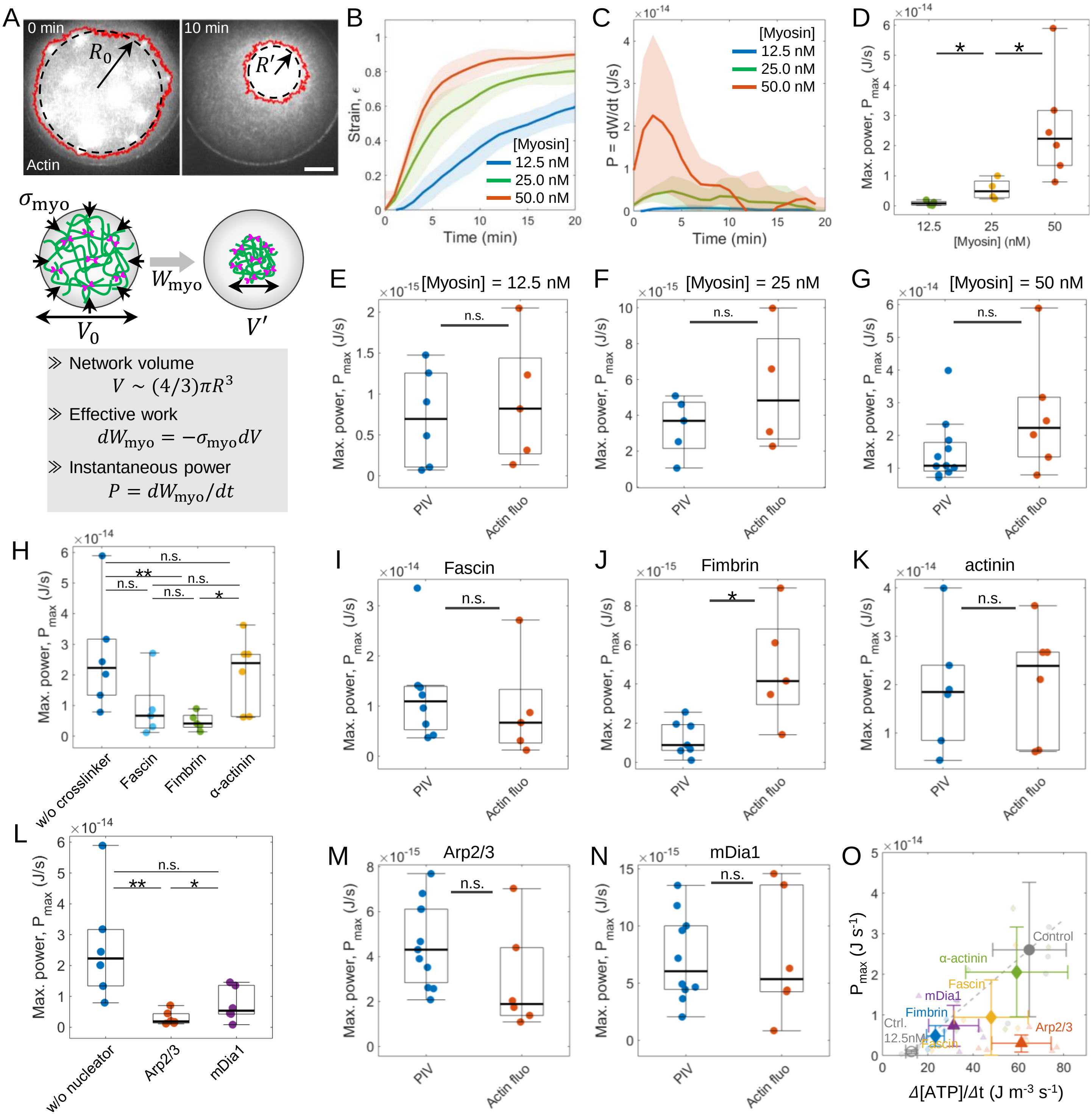

Supplementary Figure 6. Actin fluorescence-based volume change and mechanical power estimation. (**A**) The cross-sectional area of the contracting actin network is detected from the actin fluorescence image and then approximated as a circle with radius R, which is used to calculate the volume of the network, $V \sim (4/3)\pi R^3$, volumetric strain $\epsilon = -[V(t) - V_0]/V_0$, the instantaneous mechanical work dW_{myo} and power P. Scale bar is 10 µm. (**B** and **C**) Strain (B) and instantaneous power (C) of the actin network over time (n=5 droplets and N=3 independent experiments in 12.5 nM; n=4 and N=4 in 25 nM; n=6 and N=5 in 50 nM). Curves are mean±std. (**D**) Maximum instantaneous power performed by myosin. (**E** to **G**) Comparison between the maximum instantaneous power calculated from PIV-displacement field and actin fluorescence for different myosin concentration (n=6 and N=3 for PIV at 12.5 nM; n=5 and N=3 for PIV at 25 nM; n=11 and N=10 for PIV at 50 nM). (**H**) Maximum instantaneous power in the presence of different crosslinkers estimated from actin fluorescence-based volume change (n=6 and N=5 in without crosslinkers; n=6 and N=3 in fascin; n=5 and N=3 in fimbrin; n=6 and N=3 in

α-actinin). (**I** to **K**) Comparison between the maximum instantaneous power calculated from PIV-displacement field and actin fluorescence for different crosslinkers (n=8 and N=7 in fascin PIV; n=6 and N=6 in fimbrin PIV; n=6 and N=3 in α-actinin PIV). (**L**) Maximum instantaneous power in the presence of different nucleators estimated by actin fluorescence-based volume change (n=6 and N=5 without nucleators; n=6 and N=3 in Arp2/3; n=6 and N=4 in mDia1). (**M** and **N**) Comparison between the maximum instantaneous power calculated from PIV-displacement field and actin fluorescence for different nucleators (n=10 and N=9 in Arp2/3 PIV; n=10 and N=7 in mDia1 PIV). (**O**) Scatter plot showing the maximum power vs the ATP consumption rates calculated based on actin fluorescence-based volume change. *, **, and *** represent $p<0.05$, $p<0.01$, and $p<0.001$, respectively. n.s., not significant.

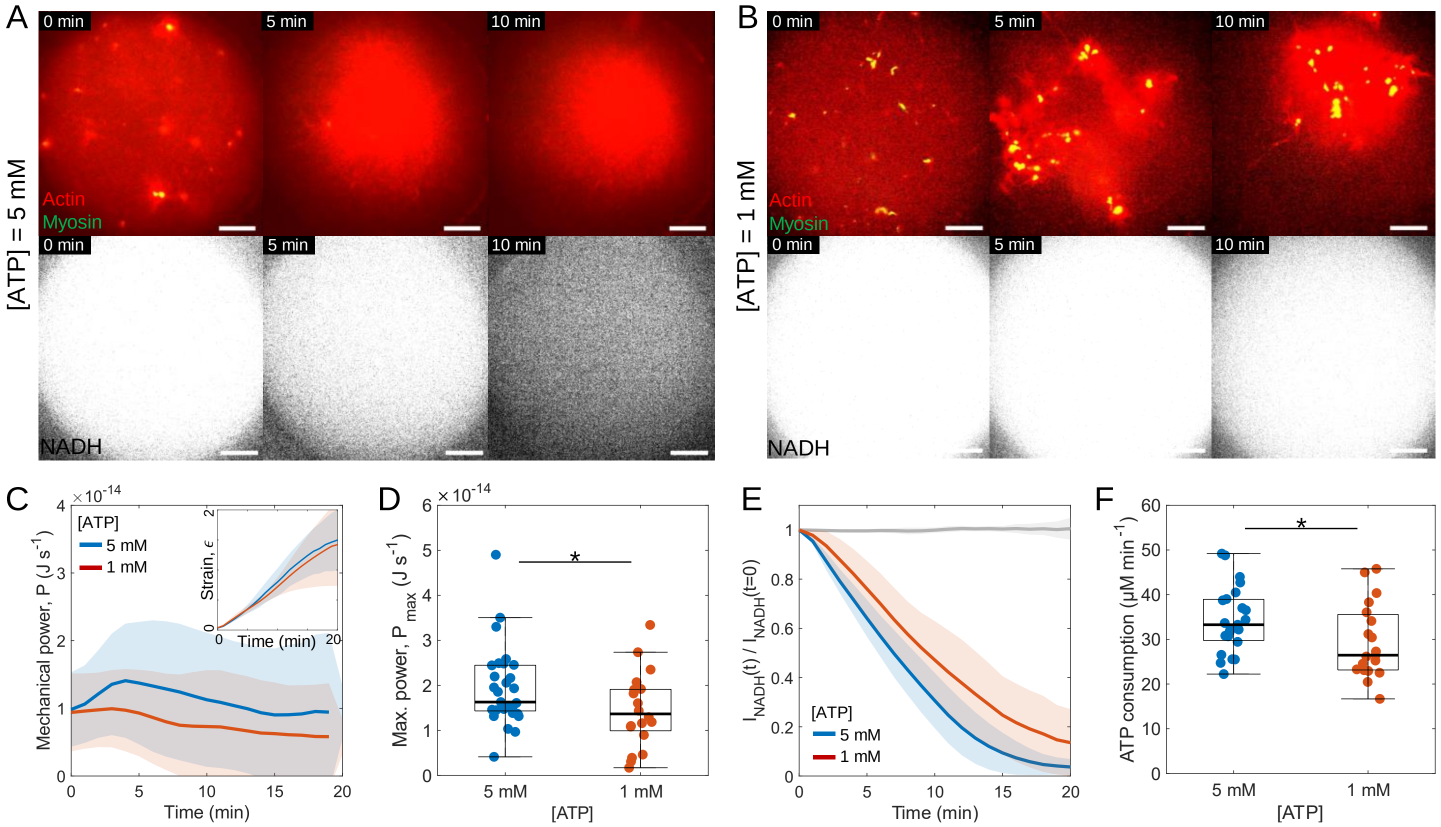

Supplementary Figure 7. ATP concentration dependence of the ATP consumption and mechanical power. (**A** and **B**) Time-lapse images showing the contraction of the actin network with ATP concentration 5 mM (A) and 1 mM (B) (actin in red, myosin in green), and the NADH fluorescence (in white). (**C**) Instantaneous power over time (n=29 droplets and N=20 independent experiments in [ATP] = 5 mM; n=20 and N=13 in [ATP] = 1 mM). Inset shows mean compressive strain over time. Curves are mean±std. (**D**) Boxplot showing the maximum instantaneous power performed by myosin extracted from (C). (**E**) NADH fluorescence over time normalized by the initial time point (n=23 and N=11 in [ATP] = 5 mM; n=19 and N=10 in [ATP] = 1 mM). (**F**) Boxplot showing the ATP consumption rate in (E). * represents $p<0.05$. Scale bars, 10 μm.

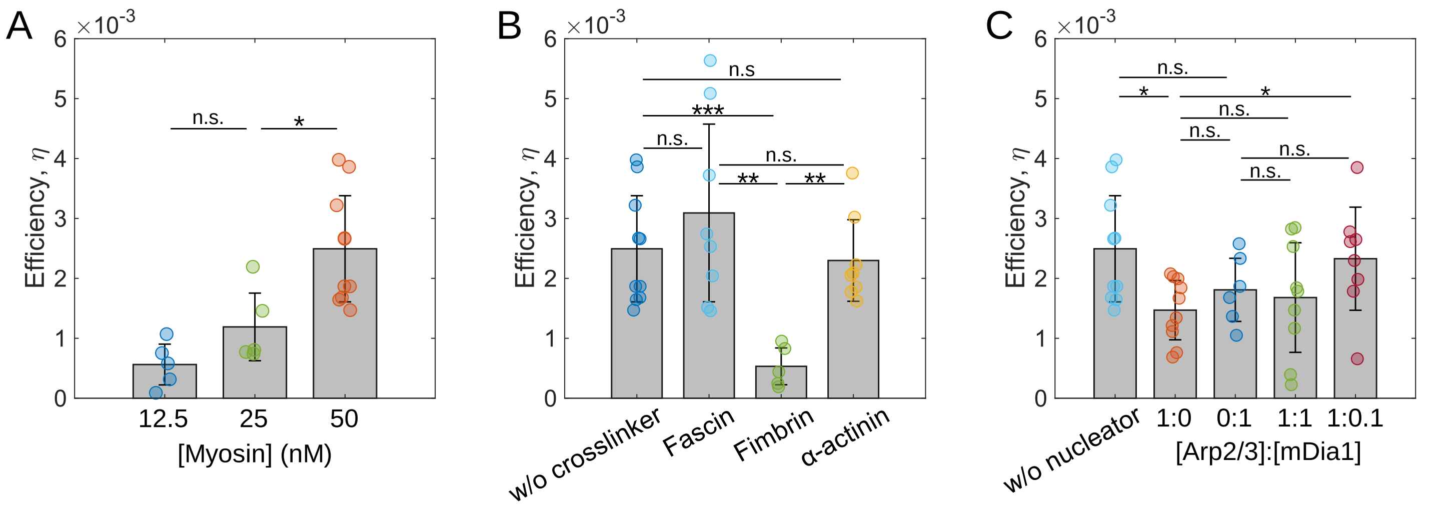

Supplementary Figure 8. The lower bound efficiency of energy conversion. The efficiency of the conversion of the free energy from ATP hydrolysis per unit time ($\Delta G_{\mathrm{ATP}}/\Delta t$) to mechanical power ($P_{\mathrm{max}}$) is estimated through the ratio of the maximum power to ATP consumption rate in a droplet, $\eta = P_{\mathrm{max}}/(\Delta G_{\mathrm{ATP}}/\Delta t)$. The ATP consumption rate is converted to the free energy of ATP hydrolysis by using the literature value of free energy released from ATP hydrolysis in physiological condition ~50-70 kJ mol^{-1} (19,20). It is noteworthy that the estimate of mechanical power in the present study represents a lower bound. Consequently, the estimated energy conversion efficiency is also a lower bound. (**A**) Barplots showing the energy conversion efficiency at different myosin concentration (n=5 droplets and N=3 independent experiments for 12.5 nM; n=3 and N=5 for 25 nM; n=10 and N=8 for 50 nM). The efficiency is larger for the higher myosin concentration, which could be because the higher myosin concentration may increase the probability of transient crosslinking within the actin network via myosin thick filaments, which may transmit contractile forces more efficiently. (**B**) Barplots showing the energy conversion efficiency in various crosslinkers (n=10 and N=8 in without crosslinkers; n=8 and N=5 in fascin; n=5 and N=3 in fimbrin; n=8 and N=5 in α-actinin). Although fimbrin-crosslinked mixed polar bundles can reduce the ATP consumption, due to significantly small mechanical power, the resulting efficiency is lower than that of the other crosslinked networks. (**C**) Barplots showing the energy conversion efficiency in various nucleator compositions (n=10 and N=8 in without nucleators; n=10 and N=9 for Arp2/3 only; n=6 and N=5 in mDia1 only; n=9 and N=6 in [Arp2/3]:[mDia1] = 1:1; n=8 and N=4 in [Arp2/3]:[mDia1] = 1:0.1). The efficiency of energy conversion is low for the Arp2/3-nucleated branched network, while the linear network nucleated by mDia1 improves the efficiency when it is mixed with the branched network. *, **, and *** correspond to p<0.05, p<0.01, and p<0.001, respectively. n.s.: not significant.

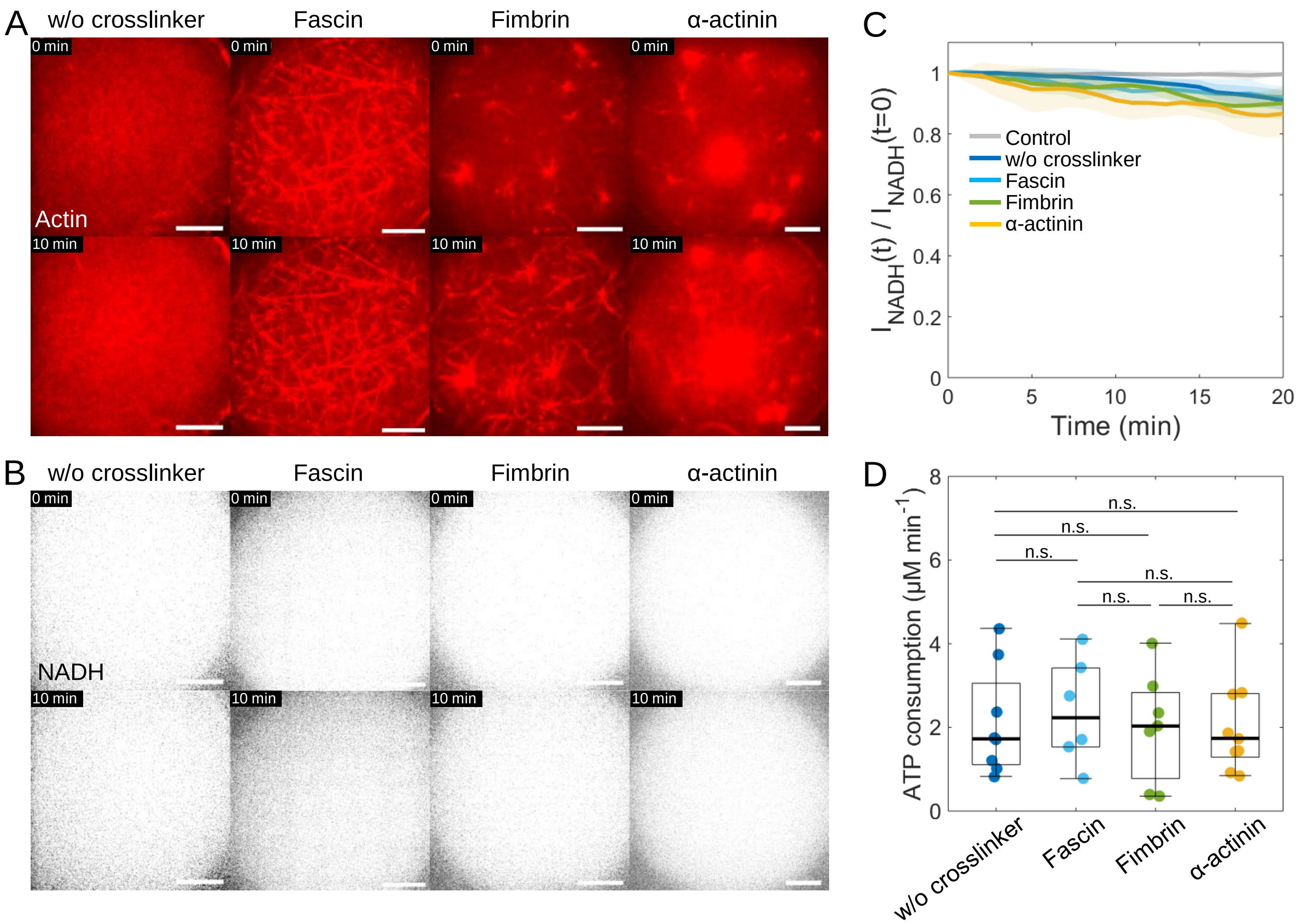

Supplementary Figure 9. ATP consumption rate of actin in the presence of actin crosslinkers. (**A** and **B**) Snapshots showing the actin fluorescence (A) and NADH fluorescence (B) within droplets containing actin and actin crosslinkers, fascin, fimbrin, or α-actinin without myosin present. (**C**) Normalized NADH fluorescence over time (n=11 droplets and N=5 independent experiments in control; n=8 and N=4 in w/o crosslinkers; n=6 and N=3 in fascin; n=7 and N=3 in fimbrin; n=9 and N=3 in α-actinin). Curves are mean±std. (**D**) Boxplot showing the ATP consumption rate in the presence of different actin crosslinkers (n=8 and N=4 in w/o crosslinkers; n=6 and N=3 in fascin; n=7 and N=3 in fimbrin; n=9 and N=3 in α-actinin). N.s., not significant. Scale bars are 10 μm.

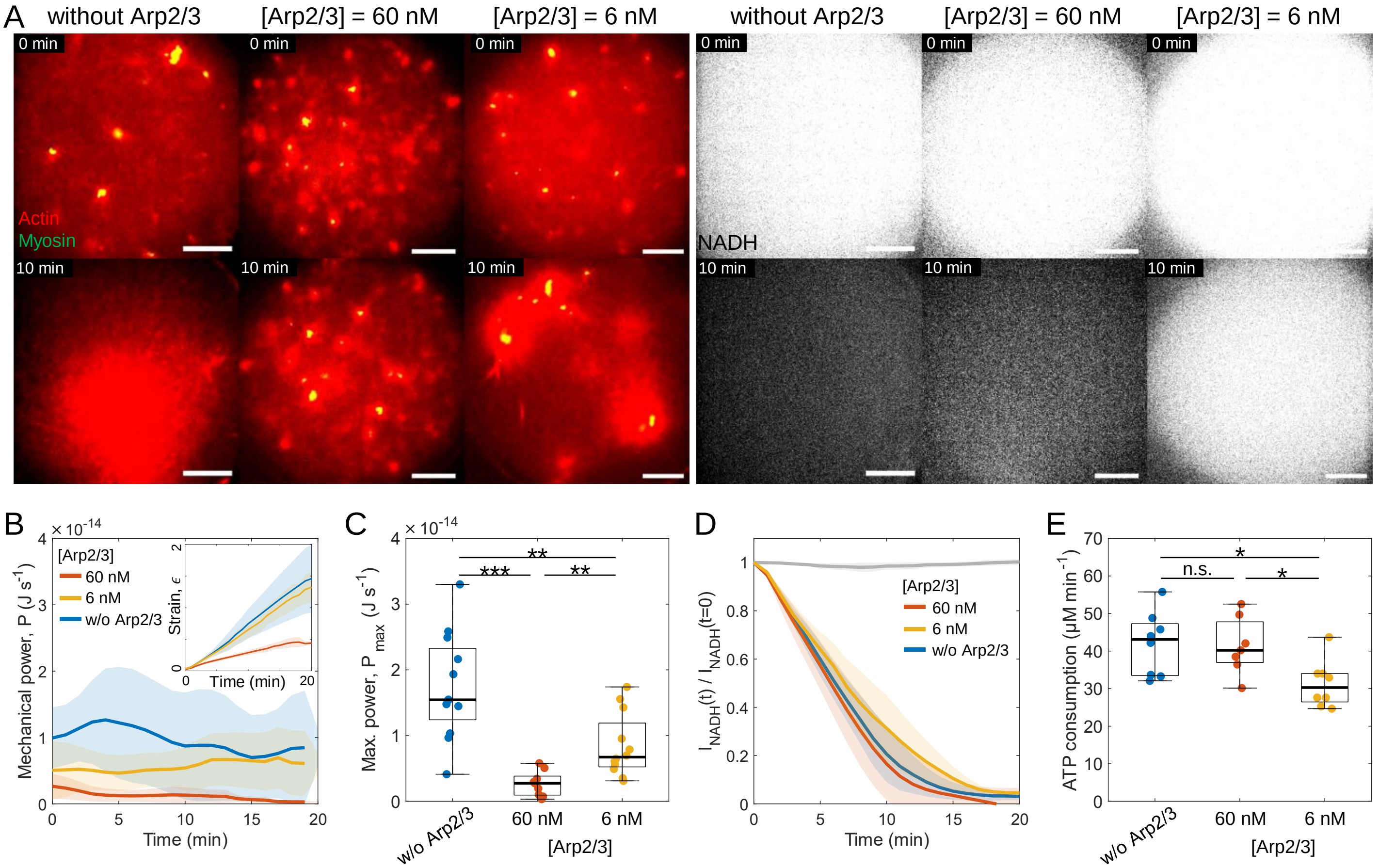

Supplementary Figure 10. Arp2/3 concentration dependence of the ATP consumption and mechanical power. (**A**) Snapshots showing the contraction of the actin network without Arp2/3, with 60 nM Arp2/3, and with 6 nM Arp2/3 (left, actin in red, myosin in green), and the NADH fluorescence (right, in white). (**B**) Instantaneous power over time (n=12 droplets and N=11 independent experiments in without Arp2/3; n=9 and N=6 in [Arp2/3] = 60 nM; n=12 and N=7 in [Arp2/3] = 6 nM). Inset shows mean compressive strain over time. Curves are mean±std. (**C**) Boxplot showing the maximum instantaneous power performed by myosin extracted from (B). (**D**) NADH fluorescence over time normalized by the initial time point (n=8 droplets and N=6 independent experiments in without Arp2/3; n=7 and N=5 in [Arp2/3] = 60 nM; n=8 and N=5 in [Arp2/3] = 6 nM). (**E**) Boxplot showing the ATP consumption rate in (D). *, **, and *** represent $p<0.05$, $p<0.01$, and $p<0.001$, respectively. n.s., not significant. Scale bars, 10 μm.

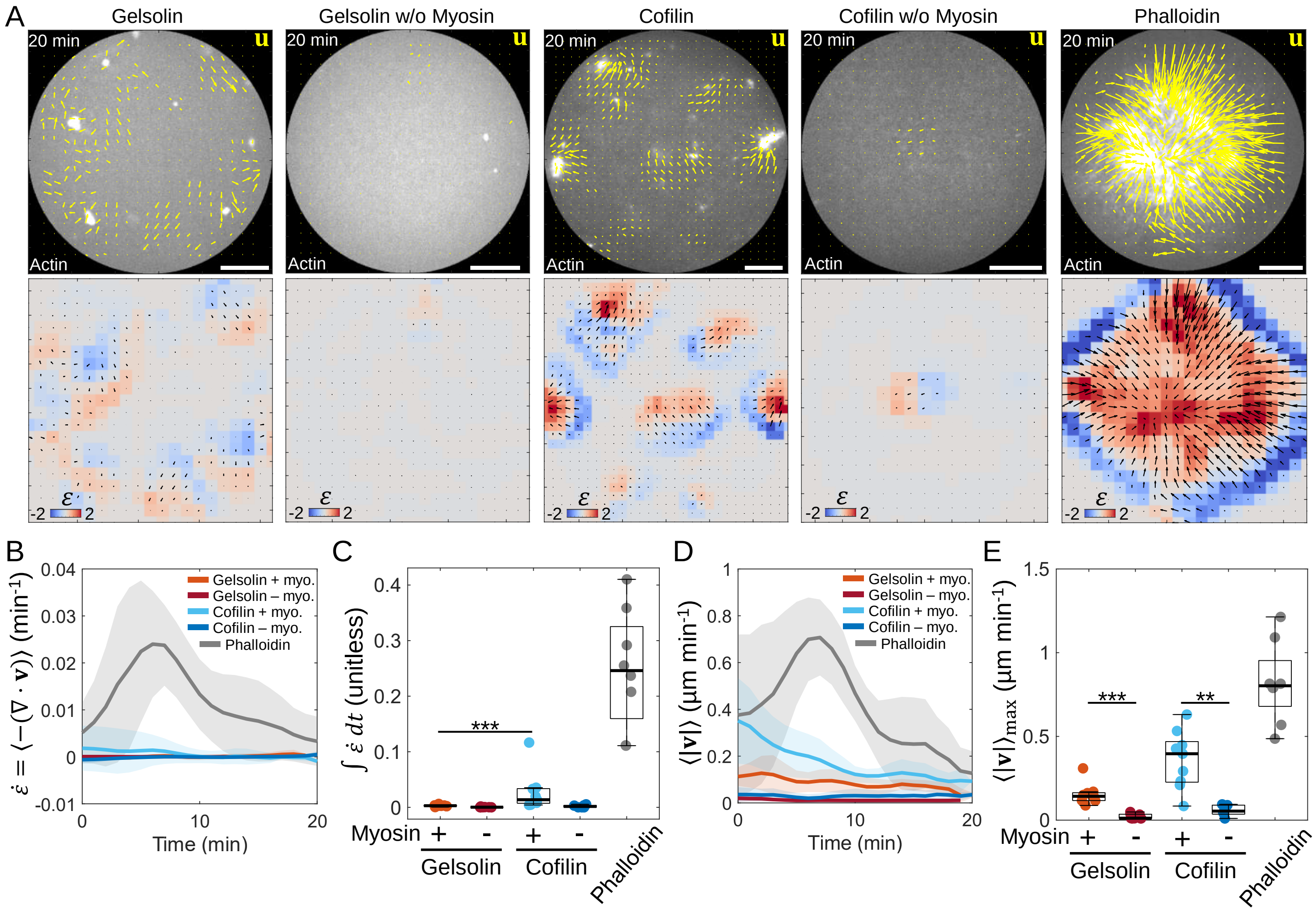

Supplementary Figure 11. The actomyosin system severed by gelsolin is in a dynamic steady-state. (**A**) PIV analysis of actomyosin network contraction in the presence of 56 nM gelsolin or 6 μM cofilin, comparing with and without myosin, and 17 μM phalloidin with myosin. Yellow vectors represent total displacement **u** over 20 min, with vector magnitudes normalized by the maximum displacement (top). The colormap represents local strain fields (bottom). (**B**) Macroscopic strain rate of the actin network ($\dot{\varepsilon} \equiv -\langle \nabla \cdot \mathbf{v} \rangle$) over time calculated from the instantaneous velocity field **v** between each frame (n=9 droplets and N=8 independent experiments in gelsolin with myosin (+) and n=8 and N=3 in gelsolin without myosin (-); n=9 and N=6 in cofilin with myosin (+) and n=6 and N=3 in cofilin without myosin (-); n=8 and N=5 in phalloidin). (**C**) Boxplots showing the total strain calculated from the integration of the strain rate in (B) over 20 min ($\int \dot{\varepsilon} dt$). The total strain is approximately 0 in the gelsolin-severed network, indicating a dynamic steady-state. By contrast, the cofilin-severed network exhibits a finite total strain due to the initial contraction and the formation of actin aggregates, suggesting that cofilin-mediated severing is insufficient to achieve a dynamic steady-state under the present experimental conditions. (**D**) Spatially averaged magnitude of velocity ($\langle |\mathbf{v}| \rangle$) over time (n=9 droplets and N=8 independent experiments in gelsolin with myosin (+) and n=8 and N=3 in gelsolin without myosin (-); n=9 and N=6 in cofilin with myosin (+) and n=6 and N=3 in cofilin without myosin (-); n=8 and N=5 in phalloidin). (**E**) Boxplots showing the maximum averaged magnitude of velocity ($\langle |\mathbf{v}| \rangle_{max}$) extracted from (D). The observed velocity in the presence of myosin is larger than that of without myosin, indicating that myosin activity induces larger fluctuations in F-actin compared to thermal fluctuations. ** and *** represent p<0.01, and p<0.001, respectively. Scale bars, 10 μm.

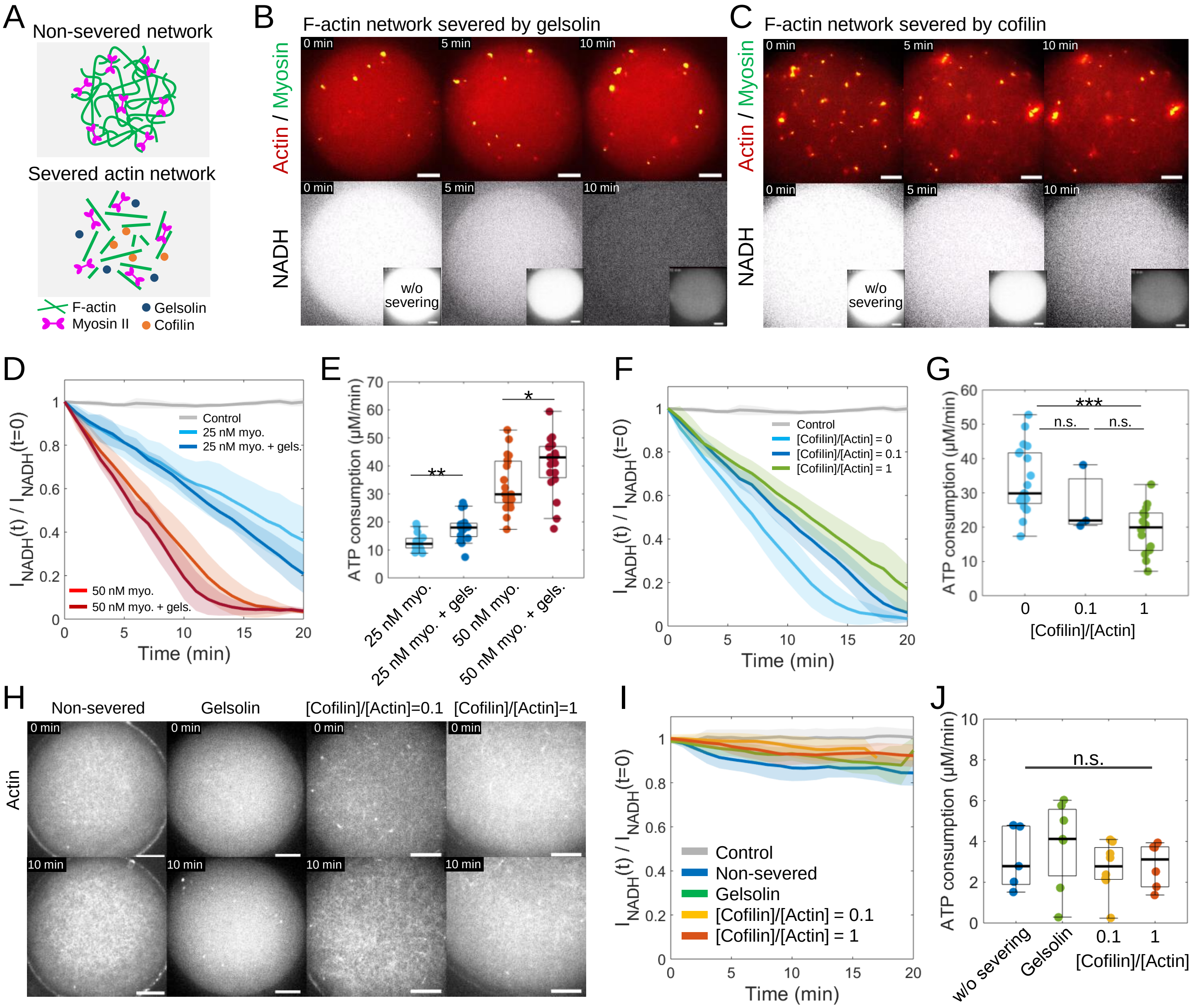

Supplementary Figure 12. The dynamic steady-state induced by gelsolin or cofilin-based severing differentially regulates ATP consumption rate. (**A**) Schematic showing the non-severed entangled actin network (top) and the freely diffusing F-actin severed by gelsolin 56 nM or cofilin 6 μM (bottom) (Movie S5). (**B** and **C**) Time-lapse images showing the actin network severed by gelsolin (B) and cofilin (C) (actin in red, myosin in green), and the NADH fluorescence (in white). (**D**) NADH fluorescence over time normalized by the initial time point (n=9 droplets and N=4 independent experiments in control only containing NADH; n=17 and N=7 at 25 nM myosin; n=17 and N=7 at 25 nM myosin with gelsolin; n=19 and N=9 at 50 nM myosin; n=20 and N=11 at 50 nM myosin with gelsolin). Curves are mean±std. (**E**) Boxplot showing the ATP consumption rate shown in D. (**F**) NADH fluorescence over time normalized by the initial time point (n=9 and N=4 in control only containing NADH; n=19 and N=9 [Cofilin]/[Actin]=0; n=3 and N=1 for [Cofilin]/[Actin]=0.1; n=14 and N=6 for [Cofilin]/[Actin]=1; 50 nM myosin for all conditions). (**G**) Boxplot showing the ATP consumption rate in (F). (**H**) Snapshots showing the droplets containing actin and severing proteins without myosin. (**I**) NADH fluorescence over time normalized by the initial time point (n=9 and N=4 in control only containing NADH; n=5 and N=3 in non-severed actin; n=8 and N=3 in gelsolin; n=9 and N=3 in [Cofilin]/[Actin]=0.1; n=6 and N=3 in [Cofilin]/[Actin]=1). (**J**) Boxplot showing the ATP consumption rate in (I). There is no significance for all combination. Scale bars, 10 μm. *, **, *** show $p<0.05$, $p<0.01$, and $p<0.001$, respectively. n.s., not significant.

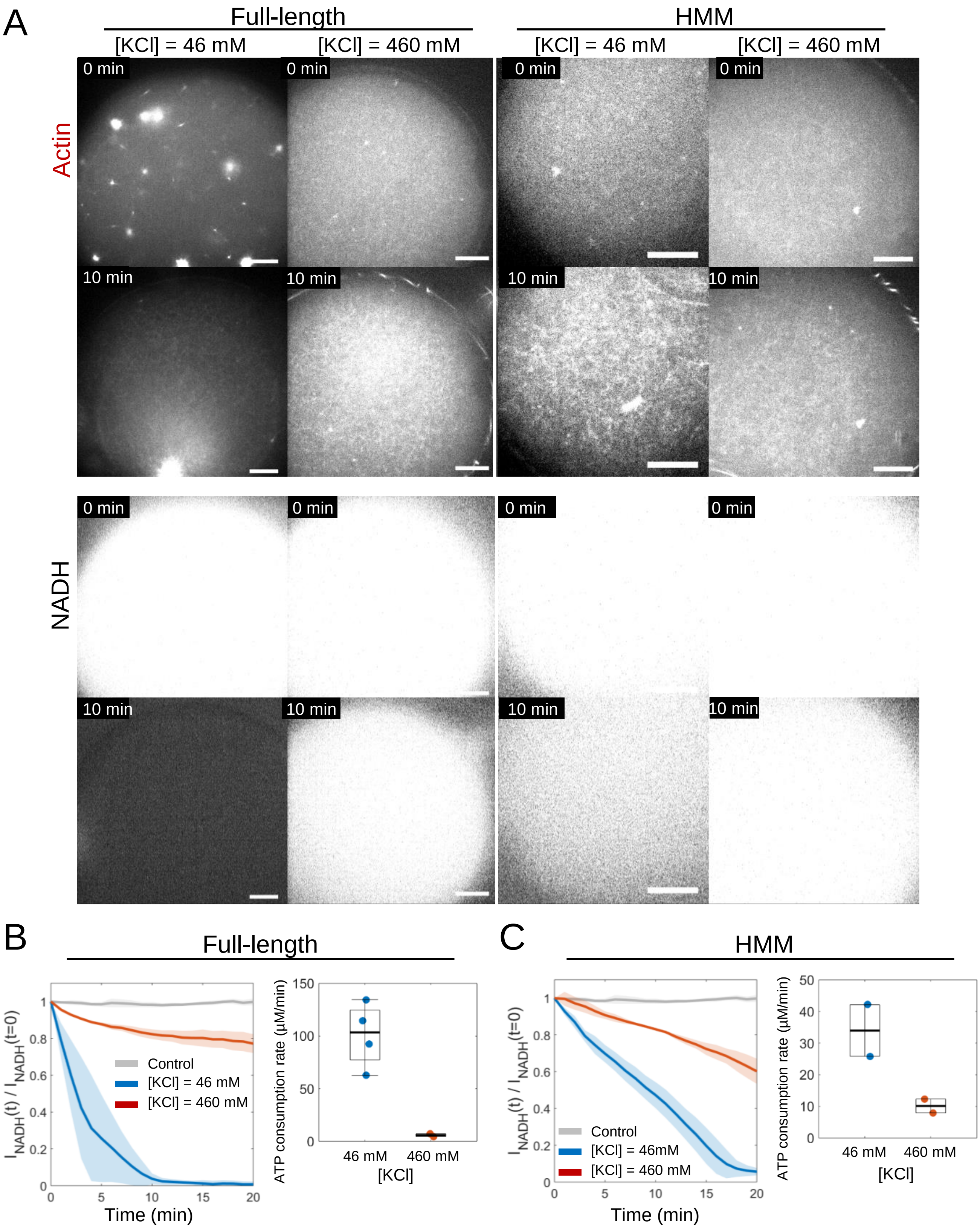

Supplementary Figure 13. ATP consumption under high salt concentration. (**A**) Snapshots showing the actin fluorescence (top) and NADH fluorescence (bottom) within droplets containing actin and full-length skeletal muscle myosin II or heavy meromyosin (HMM) for different KCl concentrations. (**B** and **C**) Normalized NADH fluorescence over time (left) and boxplot showing the ATP consumption rate for different KCl condition for droplets containing full-length (B, 0.2 μM myosin, n=4 droplets and N=2 independent experiments at 46 mM KCl; n=2 and N=1 at 460 mM KCl) and HMM (C, 0.2 μM myosin, n=2 and N=1 at 46 mM KCl; n=2 and N=1 at 460 mM KCl). Curves are mean±std. Scale bars are 10 μm.

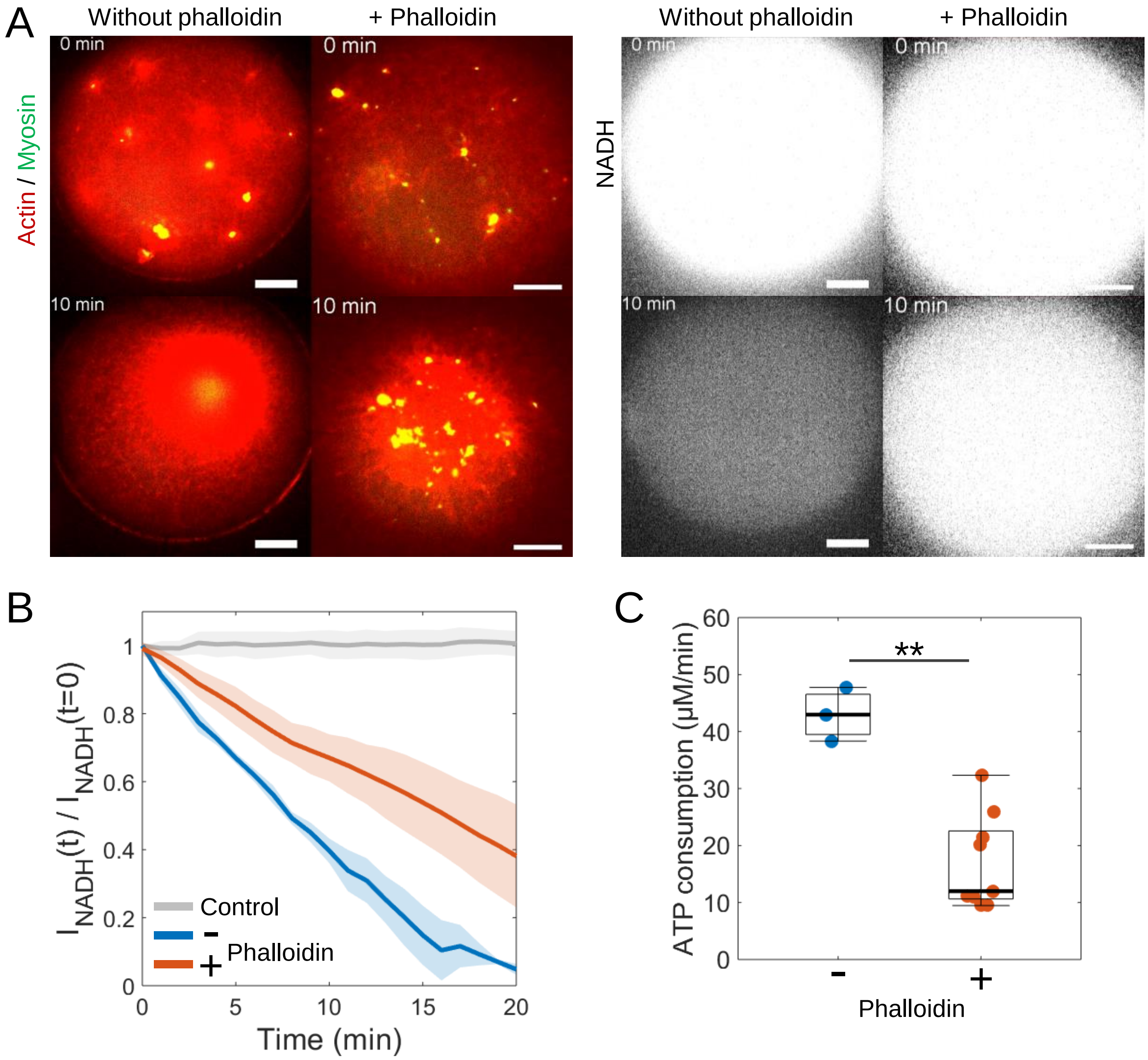

Supplementary Figure 14. ATP consumption with stabilized F-actin. (**A**) Snapshots showing the actin and myosin fluorescence (left) and NADH fluorescence (right) within droplets in the presence of 17 µM phalloidin (Movie S6). Myosin concentration is 50 nM. (**B**) Normalized NADH fluorescence over time (n=12 droplets and N=6 independent experiments in control; n=3 and N=1 in without phalloidin (-); n=9 and N=5 in with phalloidin (+)). Curves are mean±std. (**C**) Boxplot showing the ATP consumption rate in B. ** represents p<0.01. Scale bars are 10 µm.

Supplementary References

1. Gittes, F., et al., Flexural rigidity of microtubules and actin filaments measured from thermal fluctuations in shape. *J. Cell Biol.* **120**, 923-934 (1993).
2. Landau, L. D. & Lifshitz, E. M. Theory of Elasticity 2nd edn (Pergamon, Oxford, 1970).
3. Murrell, M. P. & Gardel, M. L. F-actin buckling coordinates contractility and severing in a biomimetic actomyosin cortex. *Proc. Natl. Acad. Sci. USA* **109**, 20820-20825 (2012).
4. Kasza, K. E., et al., Actin filament length tunes elasticity of flexibly cross-linked actin networks. *Biophys. J.* **99**, 1091-1100 (2010).
5. Tsuda, Y., et al., Torsional rigidity of single actin filaments and actin–actin bond breaking force under torsion measured directly by in vitro micromanipulation. *Proc. Natl. Acad. Sci. USA* **93**, 12937-12942 (1996).
6. Lenz, M. Geometrical origins of contractility in disordered actomyosin networks. *Phys. Rev. X* **4**, 041002 (2014).
7. Broedersz, C. P. & MacKintosh, F. C. Modeling semiflexible polymer networks. *Rev. Mod. Phys.* **86**, 995 (2014).
8. De La Cruz, E. M. & Gardel, M. L. Actin mechanics and fragmentation. *J. Biol. Chem.* **290**, 17137-17144 (2015).
9. Kojima, H., Ishijima, A. & Yanagida, T. Direct measurement of stiffness of single actin filaments with and without tropomyosin by in vitro nanomanipulation. *Proc. Natl. Acad. Sci. USA* **91**, 12962-12966 (1994).
10. Wollrab, V., et al. Polarity sorting drives remodeling of actin-myosin networks. *J. Cell Sci*. **132**, jcs219717 (2019).
11. Jung., W., Murrell, M. P., and Kim, T. F-actin fragmentation induces distinct mechanisms of stress relaxation in the actin cytoskeleton. *ACS Macro Lett*. **5**, 641-645 (2016).
12. Hanson, J. & Lowy, J. The structure of F-actin and of actin filaments isolated from muscle. *J. Mol. Biol*. **6**, 46-60 (1963).
13. Weirich, K. L., et al., Actin bundle architecture and mechanics regulate myosin II force generation. *Biophys. J*. **120**, 1957-1970 (2021).
14. Muresan, C. G., et al. F-actin architecture determines constraints on myosin thick filament motion. *Nat. Commun.* **13**, 7008 (2022).
15. Marcy, Y., et al., Forces generated during actin-based propulsion: A direct measurement by micromanipulation. *Proc. Natl. Acad. Sci. USA* **101**, 5992-5997 (2004).
16. Tseng, Y., et al., The bimodal role of filamin in controlling the architecture and mechanics of F-actin networks. *J. Biol. Chem*. **279**, 1819-1826 (2004).
17. Michael, K. G., et al., Structure of the actin crosslinking core of fimbrin. *Structure* 12**,** 999-1013 (2004).
18. Sakamoto, R., et al. Membrane tension induces F-actin reorganization and flow in a biomimetic model cortex. *Commun. Biol.* **6**, 325 (2023).

19. Kushmerick, M. J. & Davies, R. E. The chemical energetics of muscle contraction. II. The chemistry, efficiency and power of maximally working sartorius muscles. Appendix. Free energy and enthalpy of atp hydrolysis in the sarcoplasm. *Proc. R. Soc. Lond. B. Biol. Sci.* **174**, 315-353 (1969).
20. Milo R, Phillips R. Cell Biology by the Numbers (1st ed.) (Garland Science; 2015).
