# Supplementary material for: F-actin architecture determines the conversion of chemical energy into mechanical work
Source: Nat Commun. 2024 Apr 24;15:3444. doi: 10.1038/s41467-024-47593-x (PMC11043346; doi:10.1038/s41467-024-47593-x)
Supplement: Supplementary file 3 — Description of Additional Supplementary Files [file 41467_2024_47593_MOESM3_ESM.pdf]

Supplementary Movie 1
Time-lapse movie of actomyosin contraction and NADH fluorescence with myosin concentration of 12.5 nM, 25 nM, 50 nM, without myosin, and without actin and myosin. Actin concentration is 6 µM. Actin (red), myosin (green), NADH (white) are imaged. Scale bar is 10 µm.

Supplementary Movie 2
Time-lapse movie of actomyosin contraction and NADH fluorescence with myosin concentration of 12.5 nM, 25 nM, 50 nM. Actin concentration is 6 µM. Actin (red), myosin (green), NADH (white) are imaged. Scale bar is 10 µm.

Supplementary Movie 3
Time-lapse movie of actomyosin contraction and NADH fluorescence with 1 mM s-nitro-blebbistatin. Actin and myosin concentrations are 6 µM and 50 nM, respectively. Actin (red), myosin (green), NADH (white) are imaged. Scale bar is 10 µm.

Supplementary Movie 4
Time-lapse movie of actomyosin contraction and NADH fluorescence with 5 mM ATP, and 1 mM ATP. Actin and myosin concentrations are 6 µM and 50 nM, respectively. Actin (red), myosin (green), NADH (white) are imaged. Scale bar is 10 µm.

Supplementary Movie 5
Time-lapse movie of actomyosin contraction and NADH fluorescence with 1 µM fascin, 1 µM fimbrin, or 0.7 µM α-actinin. Actin and myosin concentration are 6 µM and 50 nM, respectively. Actin (red), myosin (green), NADH (white) are imaged. Scale bar is 10 µm.

Supplementary Movie 6
Time-lapse movie of actomyosin contraction and NADH fluorescence with 1 µM fascin, 1 µM fimbrin, or 0.7 µM α-actinin. Actin and myosin concentrations are 6 µM and 50 nM, respectively. Actin (red), myosin (green), NADH (white) are imaged. Scale bar is 10 µm.

Supplementary Movie 7
Time-lapse movie of actin and NADH fluorescence with 1 µM fascin, 1 µM fimbrin, or 0.7 µM α-actinin without myosin present. Actin concentration is 6 µM. Actin (red) and NADH (white) are imaged. Scale bar is 10 µm.

Supplementary Movie 8
Time-lapse movie of actomyosin contraction and NADH fluorescence with 300 nM Arp2/3, 300 nM mDia1, [Arp2/3]:[mDia1]=1:1, and [Arp2/3]:[mDia1]=1:0.1. Actin and myosin concentrations are 6 µM and 50 nM, respectively. Profilin concentration is 3 µM. Actin (red), myosin (green), NADH (white) are imaged. Scale bar is 10 µm.

Supplementary Movie 9
Time-lapse movie of actomyosin contraction and NADH fluorescence with 300 nM Arp2/3, 300 nM mDia1, [Arp2/3]:[mDia1]=1:1, and [Arp2/3]:[mDia1]=1:0.1. Actin and myosin concentrations are 6 µM and 50 nM, respectively. Profilin concentration is 3 µM. Actin (red), myosin (green), NADH (white) are imaged. Scale bar is 10 µm.

Supplementary Movie 10
Time-lapse movie of actomyosin contraction and NADH fluorescence with 60 nM Arp2/3, and 6 nM Arp2/3. Actin and myosin concentrations are 6 µM and 50 nM, respectively. Actin (red), myosin (green), NADH (white) are imaged. Scale bar is 10 µm.

Supplementary Movie 11
Time-lapse movie of actin and NADH fluorescence with 300 nM Arp2/3, and 300 nM mDia1 without myosin present. Actin concentration is 6 µM. Profilin concentration is 3 µM. Actin (red) and NADH (white) are imaged. Scale bar is 10 µm.

Supplementary Movie 12
Time-lapse movie of actomyosin contraction and NADH fluorescence with 56 nM gelsolin, 0.4 µM

cofilin. Actin and myosin concentrations are 6 µM and 50 nM, respectively. Actin (red), myosin (green), NADH (white) are imaged. Scale bar is 10 µm.

Supplementary Movie 13
Time-lapse movie of actomyosin contraction and NADH fluorescence with 17 µM phalloidin. Actin and myosin concentrations are 6 µM and 50 nM, respectively. Actin (red), myosin (green), NADH (white) are imaged. Scale bar is 10 µm.
